# Automatic 3D Augmented-Reality Robot-Assisted Partial Nephrectomy Using Machine Learning: Our Pioneer Experience

**DOI:** 10.3390/cancers16051047

**Published:** 2024-03-04

**Authors:** Alberto Piana, Daniele Amparore, Michele Sica, Gabriele Volpi, Enrico Checcucci, Federico Piramide, Sabrina De Cillis, Giovanni Busacca, Gianluca Scarpelli, Flavio Sidoti, Stefano Alba, Pietro Piazzolla, Cristian Fiori, Francesco Porpiglia, Michele Di Dio

**Affiliations:** 1 Division of Urology, Department of Oncology, School of Medicine, University of Turin, San Luigi Hospital, 10043 Turin, Italyfederico.piramide@gmail.com (F.P.); sabrinatitti.decillis@unito.it (S.D.C.); francesco.porpiglia@unito.it (F.P.); 2 Department of Surgery, Candiolo Cancer Institute FPO-IRCCS, 10060 Turin, Italy; 3 Romolo Hospital, 88821 Rocca di Neto, Italy; gianluca.scarpelli@gmail.com (G.S.); fla.s.10@hotmail.it (F.S.);; 4 Division of Urology, Department of Surgery, Annunziata Hospital, 87100 Cosenza, Italy; m.didio@aocs.it

**Keywords:** artificial intelligence, three-dimensional imaging, robotic surgery, renal cell carcinoma, kidney cancer, partial nephrectomy, nephron-sparing surgery

## Abstract

**Simple Summary:**

The use of 3D virtual models (3DVMs) of the kidney in an augmented-reality (AR) setting has shown the potential to improve the outcomes of a robot-assisted partial nephrectomy (RAPN). However, the use of 3DVMs is still limited to referral centers for several reasons, including the need for a dedicated assistant to be able to manually perform the virtual model’s superimposition over the operative field. In order to overcome this limitation and to improve the accuracy of the overlapping process, we developed and tested a new software, called “ikidney”, based on convolutional neural networks (CNNs). The aim of this study was to report the first pioneer series on the use of artificial intelligence through CNNs to perform an automatic AR-3DVM RAPN.

**Abstract:**

The aim of “Precision Surgery” is to reduce the impact of surgeries on patients’ global health. In this context, over the last years, the use of three-dimensional virtual models (3DVMs) of organs has allowed for intraoperative guidance, showing hidden anatomical targets, thus limiting healthy-tissue dissections and subsequent damage during an operation. In order to provide an automatic 3DVM overlapping in the surgical field, we developed and tested a new software, called “ikidney”, based on convolutional neural networks (CNNs). From January 2022 to April 2023, patients affected by organ-confined renal masses amenable to RAPN were enrolled. A bioengineer, a software developer, and a surgeon collaborated to create hyper-accurate 3D models for automatic 3D AR-guided RAPN, using CNNs. For each patient, demographic and clinical data were collected. A total of 13 patients were included in the present study. The average anchoring time was 11 (6–13) s. Unintended 3D-model automatic co-registration temporary failures happened in a static setting in one patient, while this happened in one patient in a dynamic setting. There was one failure; in this single case, an ultrasound drop-in probe was used to detect the neoplasm, and the surgery was performed under ultrasound guidance instead of AR guidance. No major intraoperative nor postoperative complications (i.e., Clavien Dindo > 2) were recorded. The employment of AI has unveiled several new scenarios in clinical practice, thanks to its ability to perform specific tasks autonomously. We employed CNNs for an automatic 3DVM overlapping during RAPN, thus improving the accuracy of the superimposition process.

## 1. Introduction

The concept of “Precision Surgery” emerged as a logical extension of the broader concept of “Precision Medicine”, with a focus on tailoring surgical approaches to individual clinical scenarios [1]. The primary objective of this approach is to minimize the impact of a surgery on the patient’s overall health by preserving maximal healthy tissues during procedures. Various strategies have been proposed to achieve this objective, spanning from preclinical interventions aimed at enhancing patient awareness and involvement in clinical decisions [2] to intraoperative adjustments of surgical techniques based on patient-specific anatomy [3,4].

In the realm of urologic surgery, multiple tools have been explored to provide surgeons with insights into the anatomical and pathological features of each patient, facilitating the development of personalized surgical plans. Among these tools, the utilization of 3D virtual reconstructions derived from standard preoperative imaging modalities, such as CT scan and MRI, has gained considerable traction in recent years. This popularity stems from its capacity to eliminate the need for surgeons to mentally construct the three-dimensional morphology of target organs and pathologies [5]. One of the most promising applications of these models lies in their ability to guide surgeons intraoperatively, revealing concealed anatomical landmarks and thereby minimizing unnecessary tissue dissection and damage [6,7,8].

Currently, the manual superimposition of three-dimensional virtual models (3DVMs) has been explored, albeit with inherent limitations, such as potential imprecisions and a reliance on operator expertise. Furthermore, the requirement of dedicated personnel for manual 3DVM overlaying significantly impacts procedural costs. Leveraging artificial intelligence (AI) presents an opportunity to address these limitations by enabling an automatic and more precise alignment of virtual models in the operative field. This advancement allows for the prompt identification of concealed anatomical targets, leading to a reduced surgical duration and improved outcomes in both the demolitive and reconstructive phases. Machine learning strategies, in particular, have shown promise for automatic 3DVM overlaying in a robot-assisted partial nephrectomy (RAPN), owing to their capacity to analyze vast amounts of video data efficiently [9].

This study aims to present our initial experience with an automatic machine-learning-based 3DVM superimposition during a robot-assisted partial nephrectomy (RAPN).

## 2. Materials and Methods

From January 2022 to April 2023, patients affected by organ-confined renal masses amenable to RAPN were enrolled.

The present study was conducted according to good clinical practice guidelines, and informed consent was obtained from all the patients. An abdominal four-phase contrast-enhanced computed tomography (CT) was obtained within 3 months before the surgery. Inclusion/exclusion criteria are reported in Table 1.

Hyper-accuracy 3D models (HA3D™) were developed by bioengineers at Medics3D (Turin, Italy). As previously detailed [8,10,11], a specific software was employed for the creation of three-dimensional virtual models (3DVMs) by processing multiphase CT images in DICOM format through acquisition and subsequent segmentation. The resultant model encompassed the kidney along with its tumor, arterial and venous branches, and the collecting system. The model, saved in .stl format, was then uploaded onto a dedicated web platform, which is accessible for visualization or download by authorized users.

For the purpose of achieving a fully automated augmented-reality (AR) intraoperative navigation, a machine-learning approach employing convolutional-neural-network (CNN) technology was employed to process live images directly from the endoscope. Each surgical intervention was carried out in collaboration with a bioengineer and the software developer.

In a preclinical context, prerecorded images of RAPN were extracted from our institutional video library. Each video was tagged, and the kidney was recognized based on its position in the abdominal cavity, considering the six possible degrees of freedom the organ may assume within the surgical field, as observed by the endoscopic camera [12]. The ResNet.50 (Residual Network) software was utilized to analyze the complex multimedia files.

**Figure 1:**
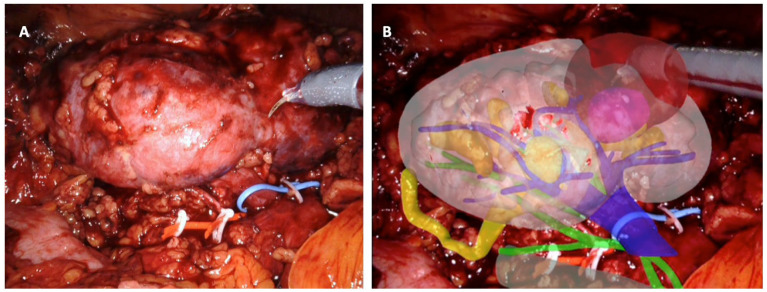
Intraoperative automatic augmented-reality co-registration process, using the software ikidney through CNN technology. (**A**) Right kidney, as it appears after renal-lodge dissection; the renal artery was tagged with a red vessel loop and the renal vein with a blue vessel loop; the tumor is partially esophytic, and it is located at the middle portion of the kidney. (**B**) Automatic overlapping of the virtual kidney over the real one, leveraging CNN technology; upper urinary tract is in yellow, arterial vessels in green, and venous vessels in blue; the tumor is in brown.

Various geometric properties of the kidney were assessed to discern its rotation, including the kidney’s center, organ size, and orientation of major and minor axes. Subsequently, a second software, ikidney, processed the images and facilitated the automatic superimposition of the 3DVM onto the intraoperative endoscopic images displayed on the robotic console. All RAPNs were performed by a highly experienced surgeon. Following standard procedure, the anterior aspect of the kidney was exposed post trocar placement and robot docking to visualize the organ. The software automatically excluded renal vessels and the collecting system, thereby limiting the degrees of freedom and generating an adjusted result by recalculating the organ’s rotation. Subsequently, the iKidney software overlaid the 3DVM onto the real-time endoscopic images (refer to Figure 1). Once an automatic alignment of the virtual model with the kidney was achieved, dissection commenced. In cases of entirely endophytic lesions, a US robotic probe was employed to verify alignment between the virtual tumor location and its real position.

Results were presented as a median (interquartile range [IQR]) or mean (standard deviation [SD]) for continuous variables and as a frequency and proportion for categorical variables. Patient demographic data, including age, body mass index (BMI), and comorbidities as classified according to the Charlson Comorbidity Index (CCI) [13], were documented. Tumor characteristics, such as the location, size, and surgical complexity as per PADUA scores, were provided [14]. Perioperative data encompassed the operative time, the management of the renal pedicle, the type and duration of ischemia, and complications. The anchoring time and the static and dynamic overlap errors of the 3D virtual models were also reported. Postoperative complications were graded according to the Clavien Dindo classification [15]. Pathological data, comprising the TNM stage and functional outcomes (serum creatinine and estimated glomerular filtration rate [eGFR]) at 3 months post-surgery, were recorded.

## 3. Results

A total of 13 patients were included in this study. The demographic and preoperative characteristics of the patients are shown in Table 2. The mean age was 67 (14) years, the mean body mass index (BMI) was 26.1 (4.2) kg/m^2^, and the median CCI was 2 (2–3). The median tumor size was 32 mm (22–43) and the median PADUA score was 9 (8–10). The average anchoring time was 11 (6–13) s Table 3. Unintended 3D-model automatic co-registration temporary failures happened in a static setting in one patient, while this happened in one patient in a dynamic setting. There was one failure; in this single case, an ultrasound drop-in probe was used to detect the neoplasm, and the surgery was performed under ultrasound guidance instead of AR guidance. The mean ischemia time was 18.1 (3.7) min for global clamping and 23.1 (4.6) min for selective clamping. Pure enucleation was performed in two cases and a violation of the collecting system was recorded in 2/8 (25%) of the surgeries. The mean operative time was 89.2 (23.3) minutes. The mean estimated blood loss (EBL) was 189.9 (135.2) ml. No major intraoperative nor postoperative complications (i.e., Clavien Dindo > 2) were recorded. Perioperative and postoperative data were collected up to 3 months after the surgery. Pathological and functional data are reported in Table 4. No positive surgical margins were recorded.

## 4. Discussion

In recent years, the use of 3DVMs has gained an increasing interest among the surgical community [16]. In fact, their different applicational options have made this technology particularly versatile. The possibility to avoid the building-in-mind process that is necessary for standard bidimensional imaging (CT scan and MRI) constitutes a potential advantage during the preoperative planning, both for young and experienced surgeons [6,17]. In urology, the use of 3DVMs has been mainly employed for prostatic and renal surgery, aiming to maximize both the functional and oncological outcomes of minimally invasive surgery [18,19].

Undoubtedly, the most promising application of this technology is represented by its use in an intraoperative setting, with the possibility to “drive” the surgeon during the most critical steps of the procedure, especially in nephron-sparing surgery. The identification of hidden anatomical targets before starting a dissection has shown to be a significant aid to optimizing this step of the procedure, reducing the risk of possible complications during a partial nephrectomy while sparing a significant amount of renal healthy tissues [7,20]. Moreover, the optimization of the clamping strategy, which aims to reduce the global ischemia rate during a dissection, may play an important role in preventing impaired renal functions after a partial nephrectomy [21]. To reach this goal, the last evolution of 3DVMs provides additional information about kidney vascularization that must be considered when a selective clamping is planned. In fact, the new “rainbow kidney” constitutes the third generation of 3DVMs, and the novelty is represented by the introduction of a renal vascularization map for every single patient, calculated from CT images, using a dedicated algorithm, as was shown in previous studies [22,23]. All of this information can be displayed on-demand in real time in the DaVinci console, thanks to the TilePro. Unfortunately, a 3DVM’s superimposition over the operative field is still realized manually, with the need of an experienced dedicated assistant. This critical issue still makes this technology not reproducible in many minor centers. In addition, a manual superimposition may lead to significant imprecisions in the overlapping process, which might affect the quality of surgical dissections or reconstructions.

The only way to substitute human hands for this task was the development of a dedicated software that was taught to recognize the target organ and to superimpose the 3DVM over it automatically, using AI. The use of this technology is currently pushing informatics over its limits, and the employment of AI could potentially revolutionize healthcare and consequently urological care [24,25,26]. We then decided to explore AI algorithms for organs and for 3DVM automatic recognitions [27,28]. Currently, a fully automatic superimposition is still not possible, but initial experiences showed promising results. The identification of local landmarks was the first mandatory step to allowing the software to correctly identify the target organ. For this reason, we decided, at first, to realize an attempt of an automatic superimposition in the setting of a robot-assisted radical prostatectomy (RARP) through deep learning, exploiting the presence of the Foley catheter, which could be easily recognized by the software [29]. At this point, we decided to translate this technology to kidney surgery, in which a new landmark identification was required. We used the whole dissected kidney as an intraoperative landmark to be identified by the software. Initially, we decided to use Indocyanine Green Fluorescence (ICG) for kidney identification, and thus a specific software, called IGNITE, was developed [30]. The automatic ICG-guided AR technology was able to anchor the 3DVM to the real kidney without human intervention with a mean registration time of 7 s. The IGNITE software correctly recognized the position and orientation of the kidney in the three spatial axes, allowing for the overlapping of the model to be maintained in a static fashion and during the movements of the camera.

The main limitation of this software was the need for an assistant to fine-tune the 3DVM overlay, especially for the rotation of the organ in the abdominal cavity. Moreover, the use of ICG may enhance some microvascular variations, which might lead to overlapping errors. Furthermore, endoscope movements and light variations sometimes resulted in technical limitations.

In order to overcome these issues, we developed a new software based on a convolutional neural network (CNN) [12], in which every pixels belonging to the kidney could be independently identified, avoiding the need for specific landmarks. The position, rotation, and dimension assumed by the organ, according to the movements of the endoscopic camera, were determined by a manual extraction and tag of still-image frames from our surgical RAPN video bank. These tagged images were used to train the neural network algorithm. Similar to the previous technology, minimal human assistance was still required at the beginning of the superimposition to fix the translation/rotation/dimension. However, this time, no exogenous substances were injected, and none of the abnormal vascular enhancements confused the algorithm.

In order to ensure maximum accuracy during virtual models’ superimpositions, other factors should be taken into account. Firstly, organic elements, such as the patient’s breath and organs’ deformations, during manipulation significantly impact the software’s performance. The global accuracy of the procedure does not only rely entirely on organ–3DVM correspondence but also on non-organic elements in the surgical field. In fact, robotic arm movements during the co-registration process may interfere with the superimposition. A new algorithm was developed by De Backer et al. to recognize any artificial elements in the surgical field, using deep learning [31]. In the future, aiming to further improve the application of this technology in real-life surgery, an integration of different AI software should be realized, preventing any case-specific variability, which may affect its reliability.

Our study is not devoid of limitations. Firstly, the high costs of software development make this technology scarcely reproducible at the moment. Moreover, a straight collaboration with engineers is still mandatory during this initial CNN testing phase, and this is not feasible in most non-university centers. In this regard, our aim is to improve the precision of our software, leading to a fully automatic overlapping process, making the surgeon totally autonomous during the 3DVM superimposition and thus significantly reducing the costs of each procedure.

## 5. Conclusions

Continuous technological development is pushing urologic surgeries to new limits. The employment of AI has unveiled several new scenarios in clinical practice, thanks to its ability to perform specific tasks autonomously. We employed AI through CNNs for an automatic 3DVM overlapping during RAPNs, improving the accuracy of the superimposition process. At the moment, a dedicated assistant is still needed to check and refine the overlapping, but future studies should aim to improve the AI software’s skills, increasing the precision of the superimposition and allowing for a total automatic process, thus improving the results of an augmented reality-guided surgery while reducing its global costs.

## Figures and Tables

**Table 1:** Inclusion/exclusion criteria.

Inclusion Criteria	Exclusion Criteria
Patients > 18 years	Anatomic abnormalities (e.g., transplanted kidney, horseshoe-shaped kidney, etc.)
Single-organ-confined renal mass cT1a	Multiple renal neoplasms
	Low-quality preoperative imaging (e.g., CT images with a slice acquisition interval of >3 mm or suboptimal enhancement)
	Imaging older than three months

**Table 2:** Perioperative variables: ASA = American Society of Anesthesiologists; BMI = body mass index; CCI = Charlson’s comorbidity index; CT = computed tomography; IQR: interquartile range; SD = standard deviation.

Variables		
Number of patients	13	
Age, yrs., mean (SD)	65 (12)	
BMI (kg/m^2^), mean (SD)	26.4 (4.4)	
CCI, median (IQR)	2 (2–3)	
ASA score, median (IQR)	2 (1–2)	
CT lesion size, mm., mean (IQR)	31 (22–41)	
Clinical stage, no. (%)	cT1a	8 (61.5)
	cT1b	3 (23.1)
	>cT2	2 (15.4)
Tumor location, no. (%)	Upper pole	4 (30.8)
	Mesorenal	6 (46.2)
	Lower pole	3 (23.1)
Tumor growth pattern, no. (%)	>50% Exophytic	2 (15.4)
	<50% Exophytic	6 (46.2)
	Endophytic	5 (38.4)
Kidney face location, no. (%)	Anterior face	8 (61.5)
	Posterior face	5 (38.4)
Kidney rim location, no. (%)	Lateral margin	9 (69.2)
	Medial margin	4 (30.8)
PADUA score, median (IQR)	8 (7–10)	
Preoperative score (mg/dL), mean SD	0.87 (0.5)	
Preoperative eGFR (ml/min), mean SD—MDRD formula	90.0 (16.4)	
Operative time (min), mean (SD)	88.6 (15.0)	
Hilar clamping, no. (%)	Global ischemia	4 (30.8)
	Selective ischemia	7 (53.8)
	Clampless	2 (15.4)
Ischemia time (min), mean (SD)	Global ischemia	19.0 (5.6)
	Partial ischemia	25.4 (10.3)
EBL (cc), mean (SD)	193.4 (120.3)	
Transfusion rate, no. (%)	1 (7.7)	
Extirpative technique, no. (%)	Pure enucleation	4 (30.8)
	Enucleoresection	9 (69.2)
Opening collecting system, no. (%)	Yes	4 (30.8)
	No	9 (69.2)
Intraoperative complications, no. (%)	0 (0)	
Postoperative complications, no. (%)	2 (15.4)	
Postoperative complications according to Clavien–Dindo, no. (%)	>2	0 (0)

**Table 3:** Automatic co-registration data. IQR = interquartile range.

Variables	
Co-registration time (s), median (IQR)	11 (6–13)
Static co-registration temporary failure, no. of patients (%)	1 (7.7)
Dynamic co-registration temporary failure, no. of patients (%)	2 (15.4)
Co-registration complete failure, no. of patients (%)	1 (7.7)

**Table 4:** Functional and pathological variables: eGFR = estimated glomerular filtration rate; SCr = serum creatinine; ISUP = International Society of Urological Pathology; IQR: interquartile range; SD = standard deviation.

Variables		
Postoperative score (mg/dL), mean (SD)	1.15 (0.71)	
Postoperative eGFR (ml/min), mean (SD)—MDRD formula	76.5 (20.1)	
Pathological stage, no. (%)	Benign	1 (7.7)
	pT1a	9 (69.2)
	pT1b	2 (15.4)
	pT3a	1 (7.7)
Pathological size (mm), mean SD	3.9 (21.6)	
Positive surgical margin rate, no. (%)	0 (0)	
Histopathological findings, no. (%)	Clear cell carcinoma	9 (69.2)
	Papillary	2 (15.4)
	Chromophobe	1 (7.7)
	Oncocytoma	1 (7.7)
ISUP grade, no. (%)	1	4 (30.8)
	2	7 (53.8)
	3	1 (7.7)
	Not applicable	1 (7.7)

## Data Availability

The data that support the findings of this study are available on request from the corresponding author [AP].

## Abbreviations

3D: three-dimensional; CT: computed tomography; MRI: magnetic resonance imaging; 3DVM: three-dimensional virtual models; AI: artificial intelligence; RAPN: robot-assisted partial nephrectomy; HA3D: hyper-accuracy 3D models; CNN: convolutional neural network; IQR: interquartile range; SD: standard deviation; CCI: Charlson comorbidity index; eGFR: estimated glomerular filtration rate; BMI: body mass index; EBL: estimated blood loss; ASA: American Society of Anesthesiologists; SCr: serum creatinine; ISUP: International Society of Urological Pathology; MDRD: modification of diet in renal disease; ISUP: International Society of Urological Pathology; ICG: indocyanine green.
